# Supplementary figures and images for: Gene Co-expression Is Distance-Dependent in Breast Cancer
Source: Front Oncol. 2020 Jul 24;10:1232. doi: 10.3389/fonc.2020.01232 (PMC7396632; doi:10.3389/fonc.2020.01232)

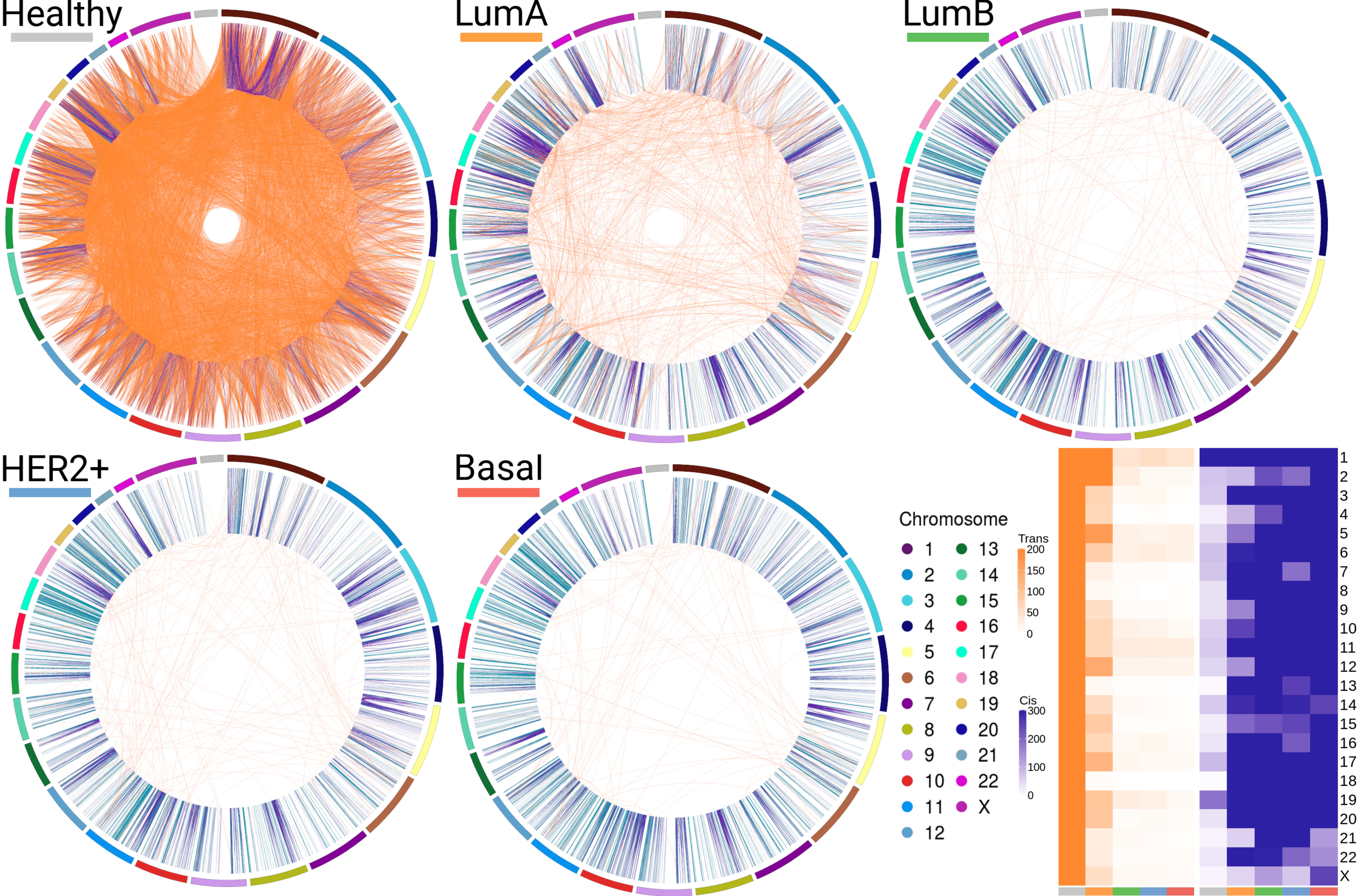

Supplement: Supplementary Figure 1 — Circos plots contain the most significant interactions for each phenotype (P < 1e−8). Orange lines join genes from different chromosomes; meanwhile purple and blue lines take account for intra-chromosomal (cis-) interactions. External circles indicate the chromosome band. Bottom right panel is a heatmap representing the number of cis- and trans- interactions per chromosome for each subtype. [file Image_1.pdf]

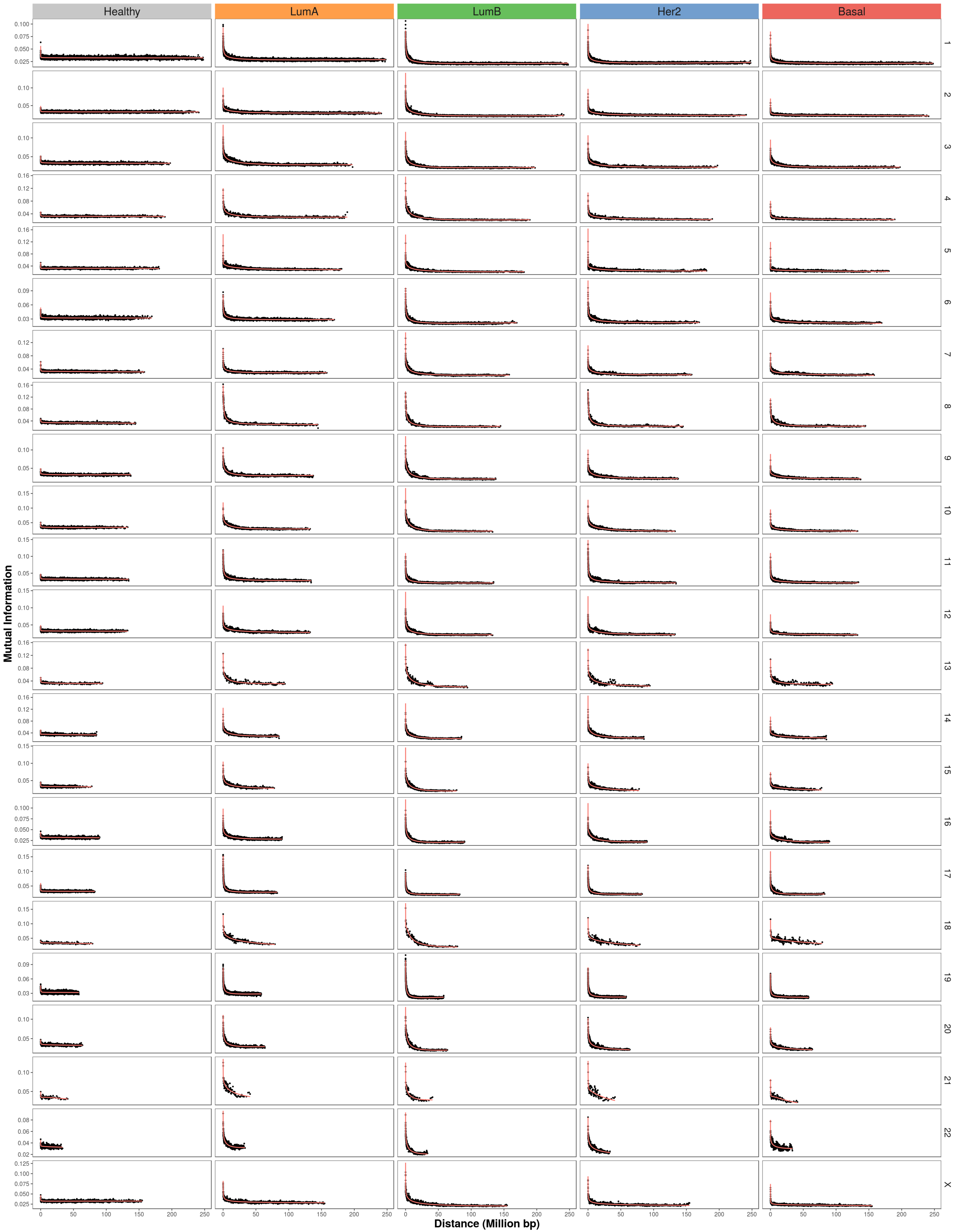

Supplement: Supplementary Figure 2 — Scatter plots showing the strength of cis- interactions with respect to physical distance in all chromosomes for the five phenotypes. [file Image_2.pdf]

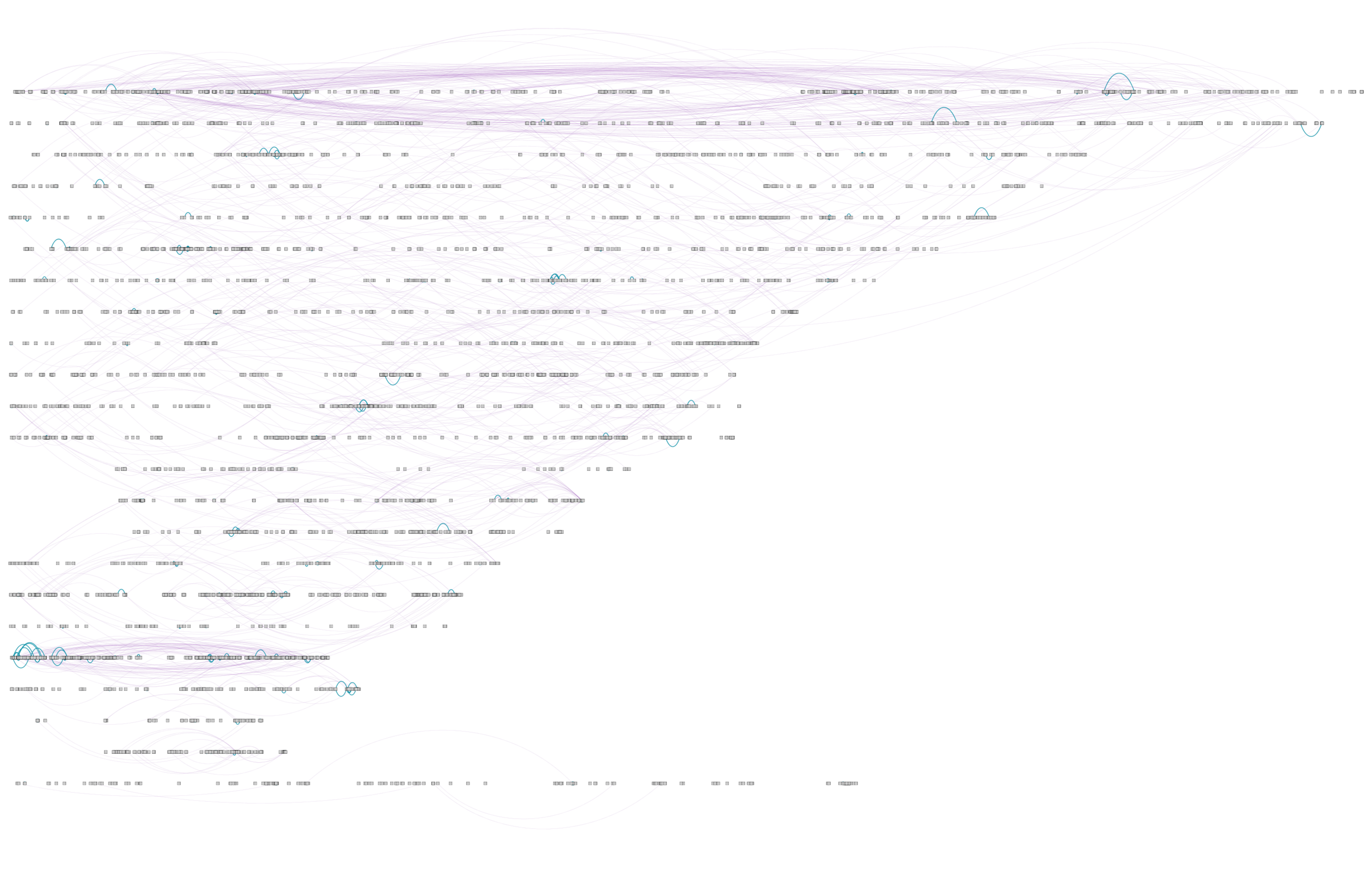

Supplement: Supplementary Figure 3 — cis- interactions for healthy network. Blue edges are intra cytoband, meanwhile inter cytoband interactions are colored in purple. [file Image_3.pdf]

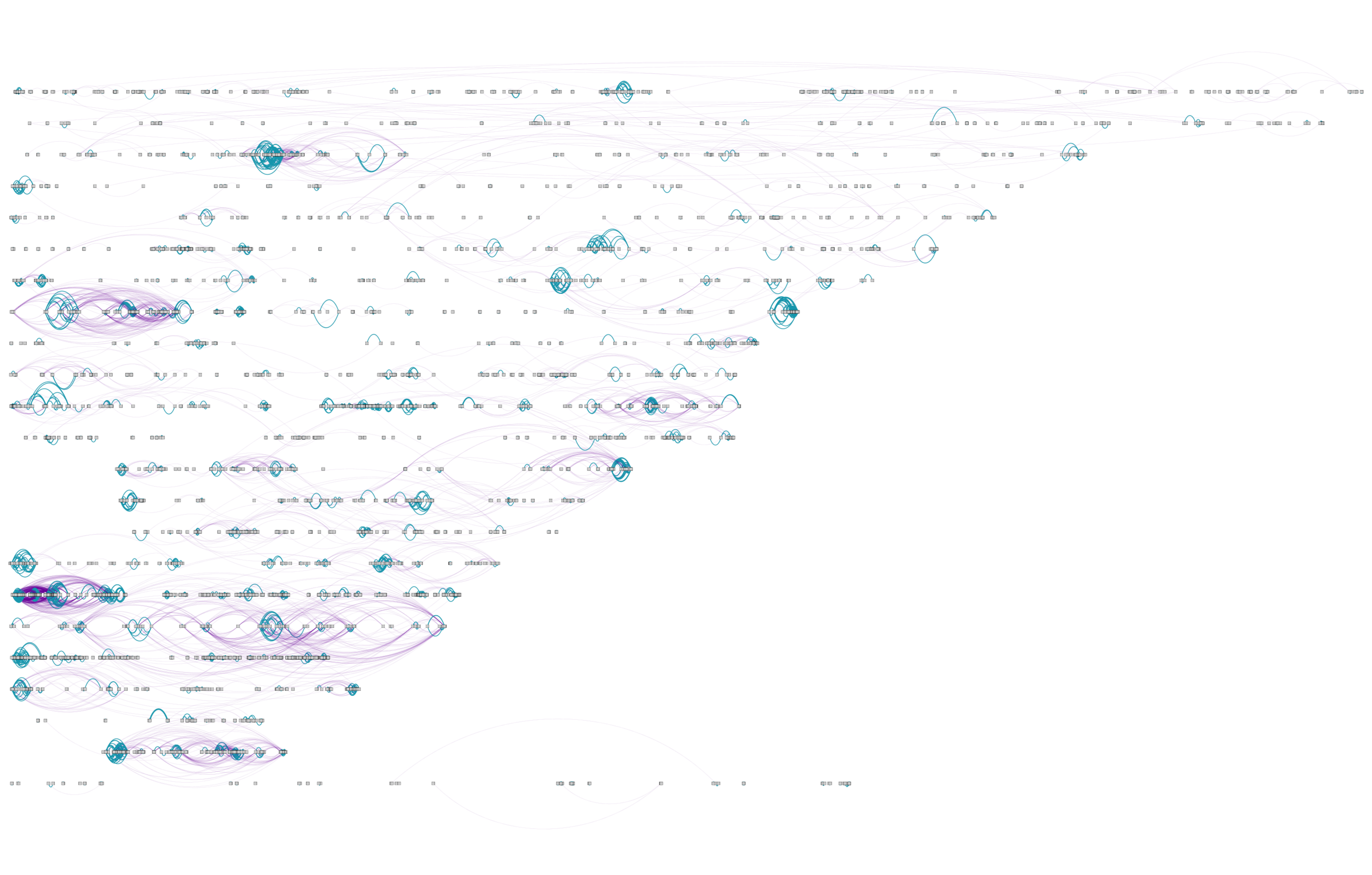

Supplement: Supplementary Figure 4 — cis- interactions for Luminal A network. [file Image_4.pdf]

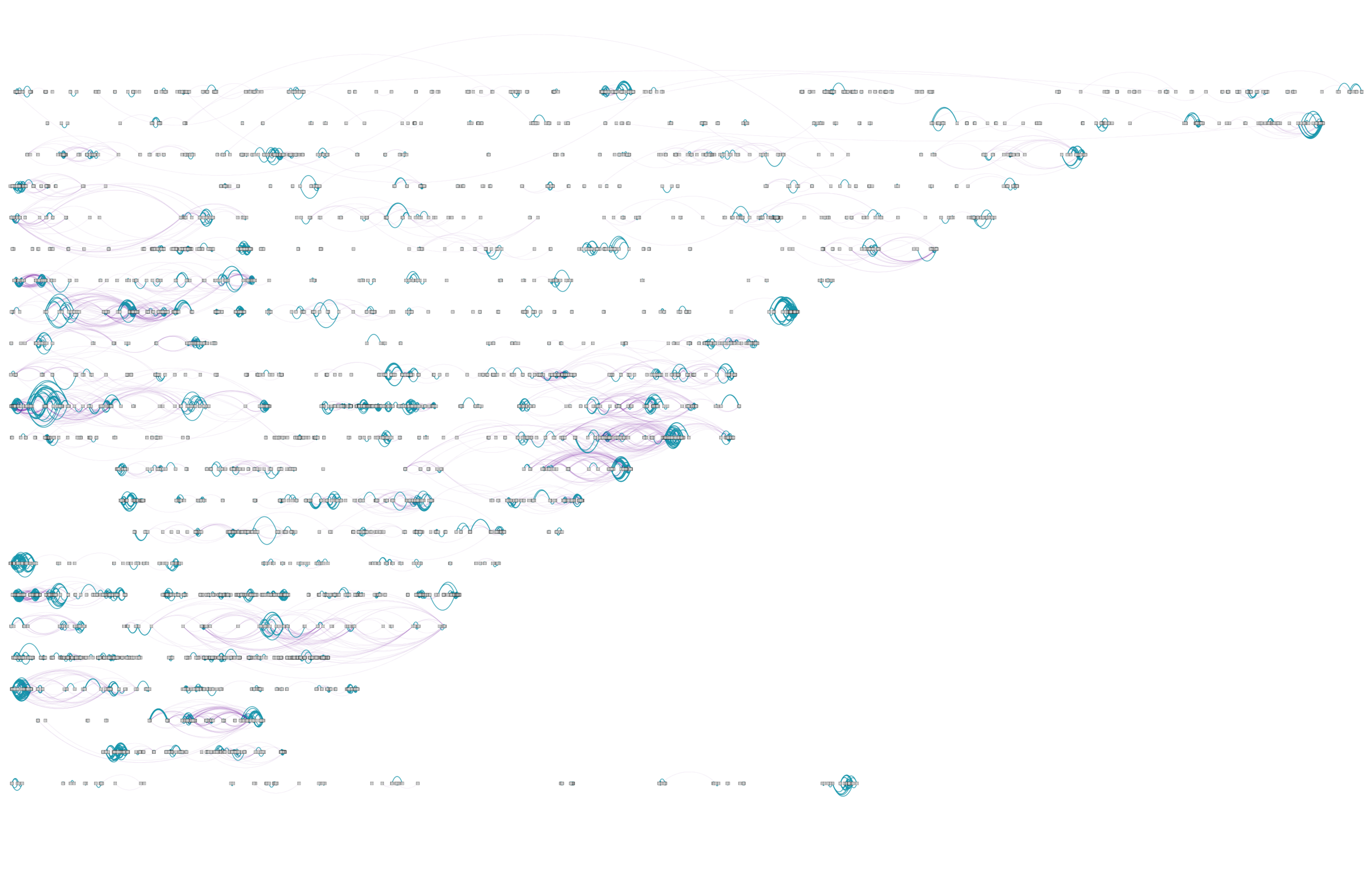

Supplement: Supplementary Figure 5 — cis- interactions for Luminal B network. [file Image_5.pdf]

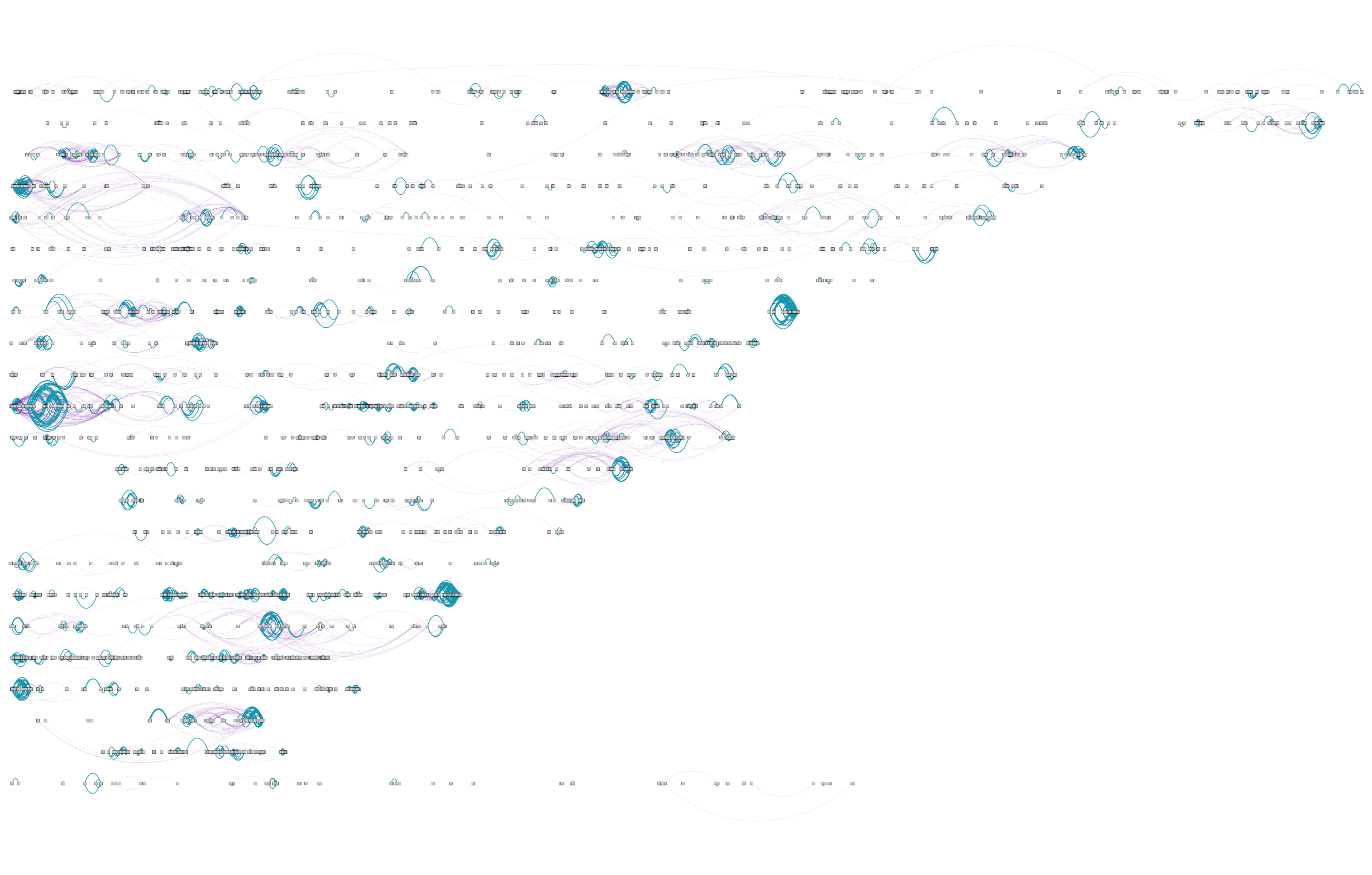

Supplement: Supplementary Figure 6 — cis- interactions for HER2+ network. [file Image_6.pdf]

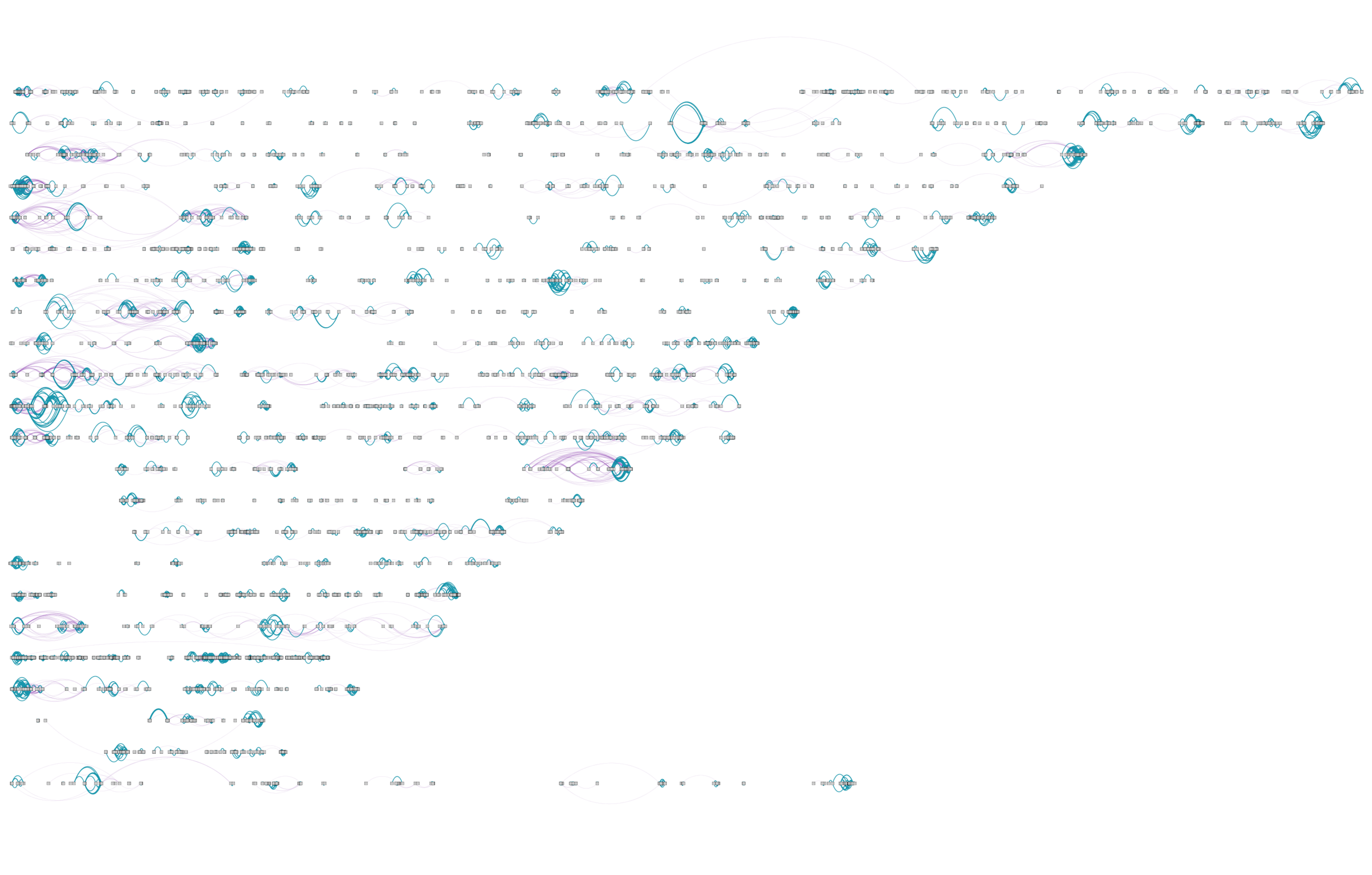

Supplement: Supplementary Figure 7 — cis- interactions for Basal network. [file Image_7.pdf]
