# Supplementary material for: Gene Co-expression Is Distance-Dependent in Breast Cancer
Source: Front Oncol. 2020 Jul 24;10:1232. doi: 10.3389/fonc.2020.01232 (PMC7396632; doi:10.3389/fonc.2020.01232)
Supplement: Supplementary Table 1 — Type-III ANOVA table results for the Intra-chromosomal regulation decay model. The ANOVA table tested the dependence of Mutual Information (MI) values using a linear model including a fourth-order polynomial for the distance (D) between gene-pairs, accounting independent intercepts for each chromosome (Chr), breast cancer subtype and also for their corresponding polynomial terms. The model was fitted in the natural logarithm (Ln) scale for both MI and D. Significance codes: 0; “***”0.001; “**”0.01; “*”0.05; “.”0.1; “”1. [file Table_1.pdf]

Table S1

Table 1: Type-III ANOVA table results for the Intra-chromosomal regulation decay model. The ANOVA table tested the dependent of the Mutual Information (MI) values using a linear model including a fourth-order polynomial for the distance (D) between gene-pairs, accounting independent intercepts for each chromosome (Chr), breast cancer subtype and also for their corresponding polynomial terms. The model was fitted in the natural logarithm (Ln) scale for both MI and D. Significance codes: 0 '***' 0.001 '**' 0.01 '*' 0.05 '.' 0.1 ' ' 1.

Model effect	Sum of Squares	Degree of Freedom	F value	Pr (>F)	Significance code
(Intercept)	0.01	1	3.9996	0.045	*
Subtype	0.12	4	9.0704	0.000000254	***
Chromosome (Chr)	0.46	22	6.247	<2.2e-16	***
Ln(Distance)	0.01	1	3.4926	0.062	.
$\mathrm{Ln(Distance)}^2$	0.01	1	3.8051	0.051	.
$\mathrm{Ln(Distance)}^3$	0.01	1	4.0679	0.044	*
$\mathrm{Ln(Distance)}^4$	0.01	1	4.291	0.038	*
Subtype:Chr	10.51	88	35.3659	<2.2e-16	***
Subtype: Ln(D)	0.13	4	9.689	7.83e-08	***
Chr: Ln(D)	0.52	22	6.9751	<2.2e-16	***
Subtype: $\mathrm{Ln(D)}^2$	0.14	4	10.2293	0.000000028	***
Chr: $\mathrm{Ln(D)}^2$	0.55	22	7.427	<2.2e-16	***
Subtype: $\mathrm{Ln(D)}^3$	0.15	4	10.7596	1.02e-08	***
Chr: $\mathrm{Ln(D)}^3$	0.57	22	7.7224	<2.2e-16	***
Subtype: $\mathrm{Ln(D)}^4$	0.15	4	11.2843	3.73e-09	***
Chr: $\mathrm{Ln(D)}^4$	0.59	22	7.9382	<2.2e-16	***
Subtype:Chr: Ln(D)	12.21	88	41.112	<2.2e-16	***
Subtype:Chr: $\mathrm{Ln(D)}^2$	13.74	88	46.2488	<2.2e-16	***
Subtype:Chr: $\mathrm{Ln(D)}^3$	15.07	88	50.7194	<2.2e-16	***
Subtype:Chr: $\mathrm{Ln(D)}^4$	16.22	88	54.5922	<2.2e-16	***
Residuals	981.23	290646			
